# Effect of single tablet regimen on prescription trends for treatment-naïve patients with HIV/AIDS in Korea

**DOI:** 10.1038/s41598-022-06005-0

**Published:** 2022-02-07

**Authors:** Kyung Sun Oh, Gi Hyeon Seo, Hee Kyoung Choi, Euna Han

**Affiliations:** 1 College of Pharmacy, Yonsei Institute of Pharmaceutical Sciences, Yonsei University, 162-1 Songdo-Dong, Yeonsu-Gu, Incheon, Republic of Korea; 2 Department of Pharmacy, Inha University Hospital, Incheon, Republic of Korea; 3 Health Insurance Review and Assessment Service, Seoul, Republic of Korea; 4 National Insurance Service Ilsan Hospital, Gyeonggi, Republic of Korea

**Keywords:** Health care, Epidemiology

## Abstract

Single-tablet regimens (STRs) should be considered for patients with HIV/AIDS to increase medication compliance and improve clinical outcomes. This study compared variations in the prescription trends between STRs and multiple-tablet regimens (MTRs) for treatment-naïve patients with HIV/AIDS after the approval of the new STRs, a proxy indicator for improvement in medication adherence. The medical and pharmacy claim data were retrospectively obtained from the Health Insurance Review and Assessment service, which contains basic information on the patients’ sociodemographic characteristics and treatment information for the entire Korean population. From 2013 to 2018, a total of 6737 patients with HIV/AIDS were included. Most patients were men (92.8%, n = 6251) and insured through the National Health Insurance (95.1%, n = 6410). The mean number of pills in their antiretroviral treatment regimens decreased from 2.8 ± 1.2 in 2013 to 1.2 ± 1.0 in 2018. After the first STR (EVG/c/TDF/FTC) was approved in 2014, prescription transitions from MTR to STR were observed among more than 38% of patients. In 2018, most treatment-naïve patients were prescribed STRs (91.2%). There was a time lag for STR prescription trends in non-metropolitan hospitals compared with those in metropolitan cities. Our data provide a valuable perspective for evaluating ART regimen prescription patterns on a national scale.

## Introduction

Over the 30 years since the human immunodeficiency virus (HIV)/acquired immunodeficiency syndrome (AIDS) was first recognized, the HIV/AIDS epidemic has remarkably transitioned from a fatal disease to a chronic illness with patients living with HIV/AIDS^1^. This achievement was made possible in part thanks to the Joint United Nations Program on HIV/AIDS^2^. The goal of the program, called the 90–90–90 target, was to diagnose 90% of all HIV-positive persons, provide antiretroviral therapy (ART) for 90% of those diagnosed with HIV/AIDS, and achieve viral suppression for 90% of patients on medication by 2020^2^.

The key to viral suppression is maintaining a potent and sustainable regimen to which people living with HIV/AIDS can easily adhere. In this sense, guidelines from the United States Department of Health and Human Services and the European AIDS Clinical Society recommend ART with combination regimens and emphasize adherence to the ART for all individuals infected with HIV/AIDS^3,4^. Early ART regimens were based on the administration of more than 20 pills, 3 times a day. Standard ART recommendations stipulate that the regimen contains at least 3 active drugs from 2 or more classes. In most cases, this consists of partial regimens combining 2 nucleoside reverse transcriptase inhibitors (NRTIs)—zidovudine (ZDV)/lamivudine (3TC), abacavir (ABC)/lamivudine (3TC), or tenofovir (TDF)/emtricitabine (FTC)—and 1 of the following: boosted protease inhibitor (PI) (lopinavir/ritonavir), nonnucleoside reverse transcriptase inhibitors (NNRTIs) (efavirenz or rilpivirine), or integrase strand transfer inhibitors (INSTIs) (raltegravir)^5,6^. Such multiple-tablet regimens are certainly not easy for adherence by patients with HIV/AIDS. Regimen characteristics, such as the complexity of the treatment regimen, long treatment duration, and frequent medication changes, have been associated with medication non-adherence^7^. Therefore, the World Health Organization (WHO) has recommended using simplified fixed-dose combination regimens for common chronic diseases, such as hypertension, tuberculosis, and HIV infection. Today, once-a-day antiretroviral treatment is recognized as the standard medication for people living with HIV/AIDS^4,8^.

The fixed-dose combinations represented by single-tablet regimens (STRs) should be considered for patients with HIV/AIDS to increase medication compliance and, consequently, improve their clinical outcomes^9,10^. The first STR that combined TDF, FTC, and efavirenz (EFV) became available globally in 2006. Many medical professionals believed that the STRs would not only reduce the patients’ pill burden and improve their quality of life but also improve medication adherence and, consequently, the probability of virologic suppression. Retention in care and virologic suppression has been achieved in the patients started on STRs^11,12^. Patients on STRs were more likely to achieve good medication adherence and had fewer hospital admissions than patients on multi-tablet regimens (MTRs)^12,13^. Increases in STR prescriptions were significantly associated with medication adherence^12,14^, which led to improved quality of life and better clinical outcomes among patients with HIV/AIDS^12,15^. Given that adherence is an essential feature in the prevention of the emergence and replication of drug-resistant strains of HIV, STRs have become an important tool to manage appropriate medication adherence^16–19^.

In Korea, the incidence of HIV/AIDS has consistently increased since the first case of HIV/AIDS infection in 1985^20^. About 1100 new patients with HIV/AIDS cases have emerged every year since 2013. To optimize ART for patients, the STR strategy was proposed in Korea, too. The first STR combining TDF, FTC, cobicistat (c), and elvitegravir (EVG) was approved in 2014 in Korea. During the 10 years between 2007 and 2018, the time when this research was conducted, there were 4 STRs available: (1) elvitegravir (EVG)/cobicistat (c)/tenofovir disoproxil fumarate (TDF)/emtricitabine (FTC) (approved in March 2014); (2) rilpivirine (RPV)/TDF/FTC (approved in December 2014); (3) dolutegravir (DTG)/ABC/3TC (approved in November 2015); and (4) EVG/C/tenofovir alafenamide fumarate (TAF)/FTC (approved in February 2017). This study compared variations in the prescription trends between STRs and MTRs for treatment-naïve patients with HIV/AIDS after the approval of the new STRs, a proxy indicator for improvement in medication adherence, using the national insurance claims data in Korea.

## Results

Table 1 shows the distributions of the variables used in the estimations. Men accounted for most of the sample (92.8%) over 6 years between 2013 and 2018. Most patients (95.1%) were insured through the National Health Insurance, with the remaining 4.9% being supported by the National Medical Aid. The mean age at index date was 38.2 ± 13.0 years, with 58.5% of patients in their 20s and 30s. Additionally, a shift of HIV/AIDS incidence toward the younger population was observed, as the proportion of patients in their 20s increased from 26.7% in 2013 to 34.8% in 2018. Patients enrolled in 2013 showed the highest proportion (25.7%) of ART prescriptions during hospital admissions, which decreased to 21.2 % in 2014 and remained below 20% from 2015 onwards. Most of the patients, 70.8–77.8% of all subjects, were treated at medical institutions located in metropolitan areas throughout the study period. Over 60% of patients were treated at tertiary general hospitals (Table 1).

**Table 1:** Baseline characteristics of the study population, n (%).

Year	2013 (n = 958)	2014 (n = 1089)	2015 (n = 1134)	2016 (n = 1214)	2017 (n = 1207)	2018 (n = 1135)	Total (n = 6737)
**Sex**							
Male	879 (91.8)	1016 (93.6)	1064 (93.8)	1130 (93.1)	1106 (91.6)	1056 (93.0)	6251 (92.8)
Female	79 (8.2)	73 (6.4)	70 (6.2)	84 (6.9)	101 (8.4)	79 (7.0)	486 (7.2)
**Insurer**							
National Health Insurance	895 (93.4)	1041 (94.2)	1078 (95.1)	1161 (95.6)	1142 (94.6)	1093 (96.3)	6410 (95.1)
National Medical Aid	63 (6.6)	48 (5.8)	56 (4.9)	53 (4.4)	65 (5.4)	42 (3.7)	327 (4.9)
**Index age group**							
Mean ± SD	39.3 ± 12.5	38.7 ± 12.9	38.3 ± 12.9	37.9 ± 13.1	37.3 ± 13.2	37.8 ± 12.9	38.2 ± 13.0
20–29	256 (26.7)	340 (34.1)	369 (32.5)	416 (34.3)	455 (37.7)	395 (34.8)	2231 (33.1)
30–39	264 (27.6)	271 (25.1)	282 (24.9)	304 (25.0)	286 (23.7)	302 (26.6)	1709 (25.4)
40–49	232 (24.2)	233 (20.7)	239 (21.1)	246 (20.3)	223 (18.5)	210 (18.5)	1383 (20.5)
50–59	145 (15.1)	175 (13.7)	166 (14.6)	157 (12.9)	158 (13.1)	152 (13.4)	953 (14.1)
≥ 60	61 (6.4)	70 (6.4)	78 (6.9)	91 (7.5)	85 (7.0)	76 (6.7)	461 (6.8)
**Antiretroviral treatment initiation**							
Inpatient	246 (25.7)	231 (21.2)	213 (18.8)	219 (18.0)	217 (18.0)	225 (19.8)	1351 (20.1)
Outpatient	712 (74.3)	858 (78.8)	921 (81.2)	995 (82.0)	990 (82.0)	910 (80.2)	5386 (79.9)
**Medical institution location**							
Metropolitan area	715 (74.6)	847 (77.8)	858 (75.7)	911 (75.0)	864 (71.6)	804 (70.8)	4999 (74.2)
Regional area	243 (25.4)	242 (22.2)	276 (24.3)	303 (25.0)	343 (28.4)	331 (29.2)	1738 (25.8)
**Medical institution type**							
Tertiary general hospital^a^	649 (67.7)	704 (64.6)	708 (62.4)	827 (68.1)	744 (61.6)	707 (62.3)	4339 (64.4)
Others^b^	309 (32.3)	385 (35.4)	426 (37.6)	387 (31.9)	463 (38.4)	428 (37.7)	2398 (35.6)

Data expressed as number (%). ^a^Tertiary general hospital is designated by the government every three years. ^b^Others include general hospital, medical clinic and regional public health center.

**Figure 1:**
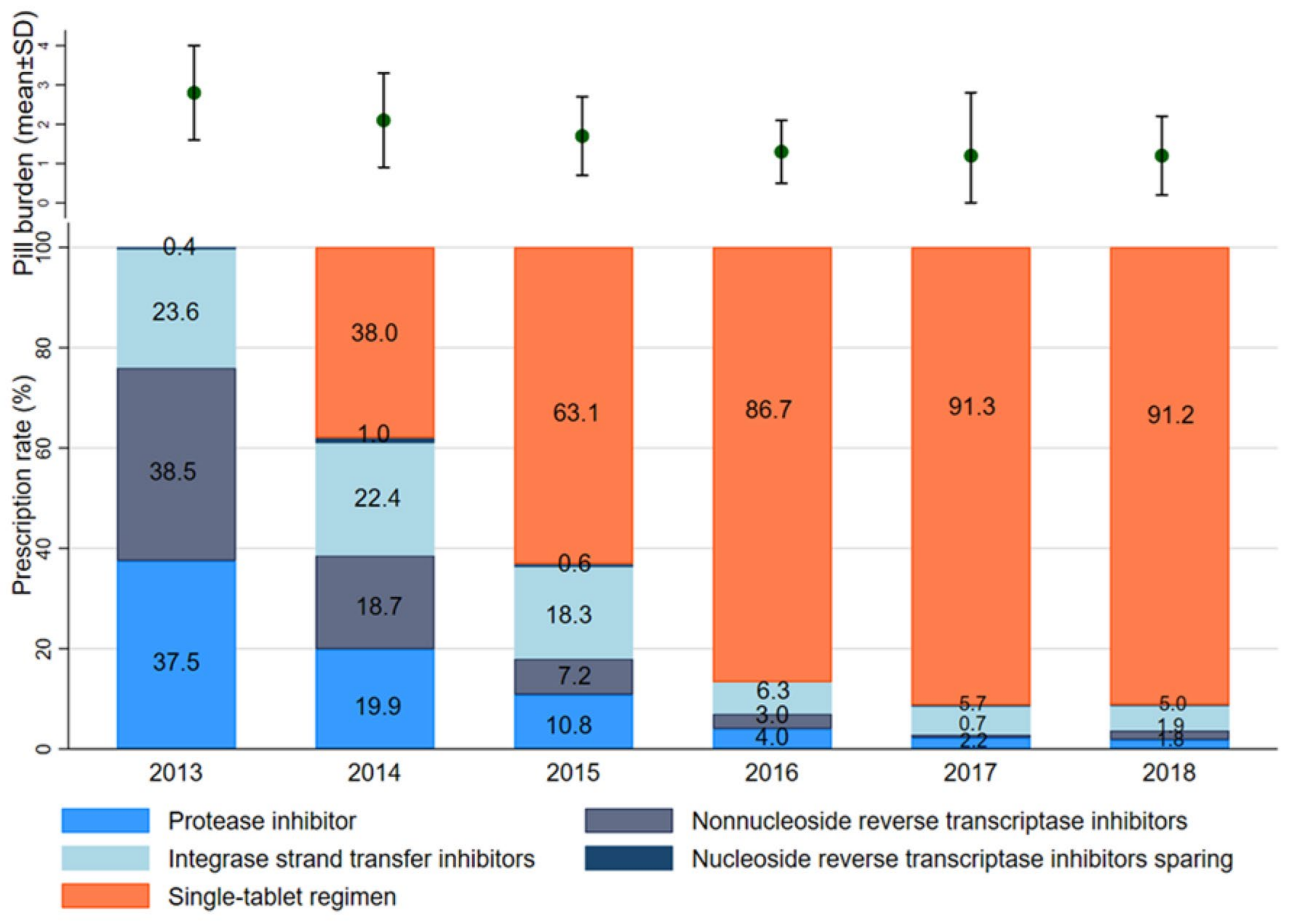
Trends in prescribed ART for treatment-naïve individuals with HIV/AIDS and change of pill burden from 2013 to 2018 in Korea. Proportions are noted in the vertical bars. ART = antiretroviral treatment; SD = standard deviation.

Since the approval of the first STR, EVG/c/TDF/FTC, in 2014, four STRs have become available sequentially (Supplementary Table 1). There were substantial differences in ART prescriptions after the first STR was approved. Overall, ART regimens have been simplified since 2014. The mean number of pills in ART regimens decreased from 2.8 ± 1.2 in 2013, just before the first STR was launched, to 1.2 ± 1.0 in 2018, after all STRs included in this analysis had been approved. Before the approval of STRs, ART prescription rates by regimen type were similar to one another; NNRTI-based regimens containing efavirenz (EFV) and boosted PIs containing lopinavir/ritonavir comprised 38.5% and 37.5% of all ART prescriptions, respectively (Fig. 1).

After the first STR (EVG/c/TDF/FTC) was approved in 2014, prescription transitions from MTR to STR were observed by more than 38% of patients, whereas NNRTI-based and PI-based MTR rates were reduced by 18.6% and 19.9%, respectively, in 2014. The proportion of patients initiating ART with STRs increased to 86.7% in 2016 after DTG/ABC/3TC was approved. In 2018, most patients who started ART were prescribed STR (91.2%, 1035 of 1135 patients) as their first HIV/AIDS treatment medication (Supplementary Fig. 2).

**Figure 2:**
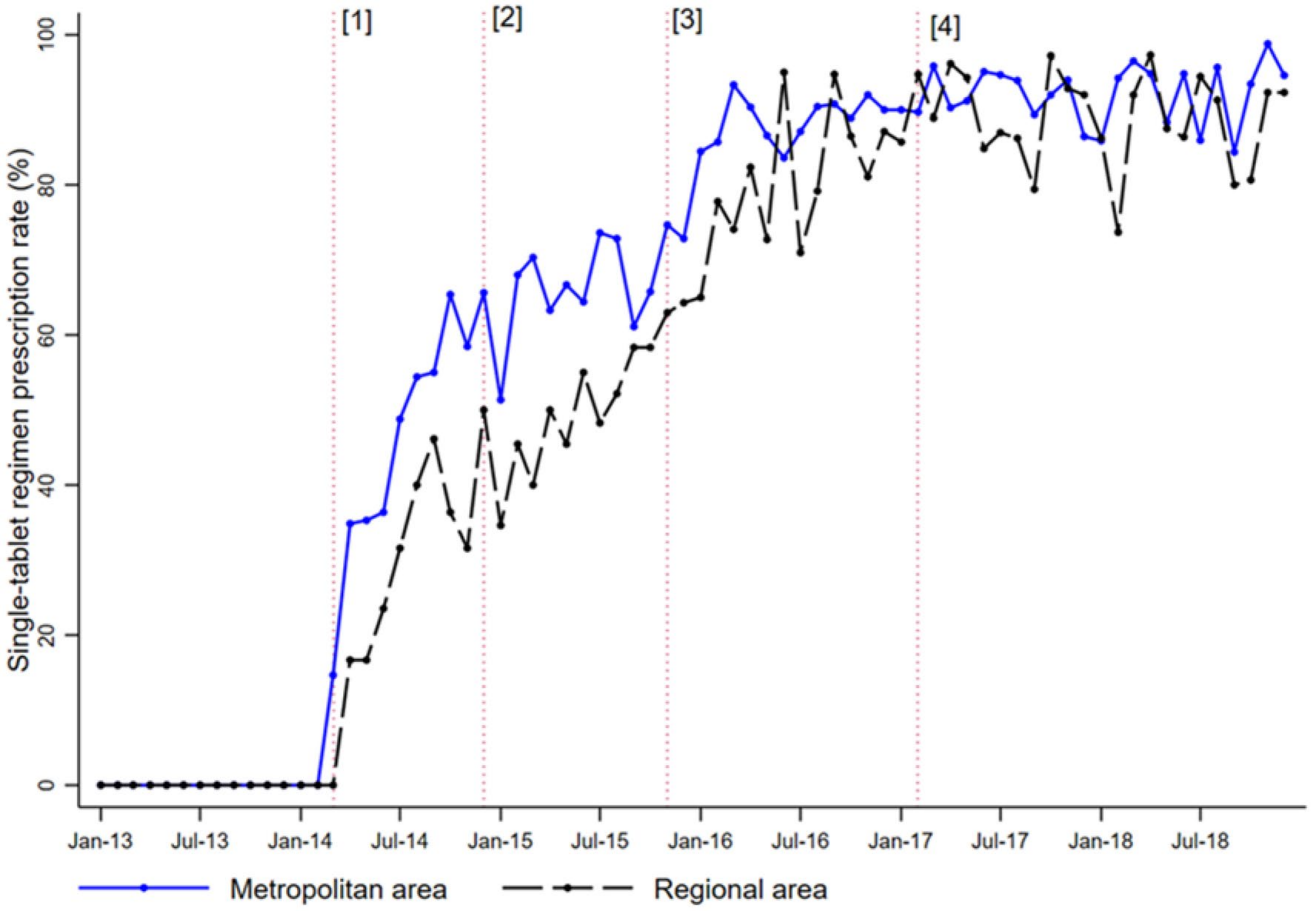
Prescription rates of single-tablet regimens from 2013 to 2018 by metropolitan area vs. regional area. Vertical lines indicate the approval date of single-tablet regimens: [1] Elvitegravir/cobicistat/tenofovir disoproxil fumarate/emtricitabine; [2] Rilpivirine/tenofovir disoproxil fumarate/emtricitabine; [3] Dolutegravir/abacavir/lamivudine; [4] Elvitegravir/cobicistat/tenofovir alafenamide fumarate/emtricitabine.

Figure 2 depicts the differences in STR versus MTR prescription trends over time by hospital location. There was a time lag for STR prescription trends in non-metropolitan hospitals compared with those in metropolitan cities. STR preference was higher in metropolitan areas than in non-metropolitan areas by more than 10% between 2014 and 2015. However, after EVG/c/TAF/FTC was approved in 2017, the prescription rates reached approximately 90% in all areas (Fig. 2).

**Figure 3:**
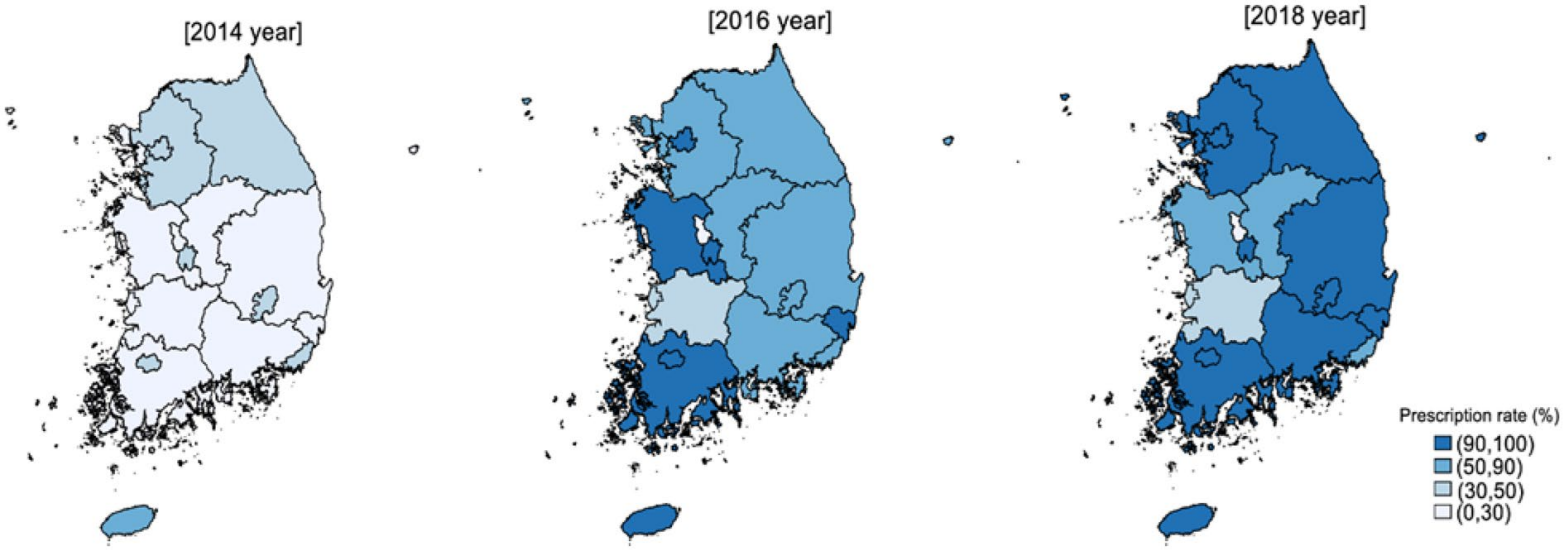
Geographic trends in the prescription rate of single-tablet regimens in South Korea. This represents percentages of single-tablet regimens for treatment- naïve individuals with HIV/AIDS by state: Seoul, Busan, Daegu, Incheon, Gwangju, Daejeon, Ulsan, Sejong-si, Gyeonggi-do, Gangwon-do, Chungcheongbuk-do, Chungcheongnam-do, Jeollabuk-do, Jellanam-do, Gyeongsangbuk-do, Gyeongsangnam-do, and Jeju-do. Maps were generated by KSO with the grmap command in STATA (StataCorp, College Station, TX, USA) and spatial data of Statistics in Korea (https://sgis.kostat.go.kr).

Figure 3 shows the biennial changes in the STR prescription rates across South Korea. The preference for STR prescriptions gradually expanded from metropolitan areas to nationwide after 2014 (Fig. 3).

## Discussion

ART prescription trends in Korea have changed dramatically in recent years since STRs were first approved. There has been a clear upward trend toward prescribing single fixed-dose combination therapy with a lower pill burden. More than 38% of treatment-naïve patients with HIV/AIDS initiated on an STR in 2014, and this proportion increased to 91% in 2018. A decline of initial ART prescriptions during hospitalization was observed, which is likely due to guideline changes by the International Antiviral Society–USA Panel and the Korean Society for AIDS, leading to ART being offered to patients with HIV/AIDS regardless of CD4 cell counts^6,21^. The preference for STR prescriptions was also observed in prior studies. STRs were the top four out of the 10 ART regimens prescribed, as per pharmaceutical benefits scheme claims data in Australia^22^. Another longitudinal study with a national cohort of United States veterans over 9 years between January 1998 and September 2016 demonstrated that the utilization of STRs has dramatically increased since their introduction in 2006, and they now account for most ART prescriptions. Efavirenz/TDF/FTC was released in 2006 as the first STR globally; therefore, it constituted 100% of STR prescriptions from 2006 to late 2011. Two STRs, DTG/ABC/3TC and EVG/c/TAF/FTC, released after efavirenz/TDF/FTC, had increasing prescription rates thereafter^23^.

Differences in clinician preferences for STR types were observed in our study. Clinicians tended to prefer INSTI-based regimens rather than NNRTI-based regimens among the STRs in Korea. EVG/c/TDF/FTC, the first approved STR in Korea, consists of 2 NRTI-based drugs (TDF and FTC) with one INSTI-based agent (EVG). In the same year, the second single-complex combination, RPV/TDF/FTC, was approved. It contains an NNRTI (RPV) with the same combination of 2 NRTIs (TDF and FTC). Notwithstanding, EVG/c/TDF/FTC, the first approved STR, was the most frequently prescribed STR from 2014 to 2015. INSTI-based regimens containing the novel ingredients DTG or EVG replaced previously preferred MTRs. EVG/c/TDF/FTC has been replaced by EVG/c/TAF/FTC since 2017. Notably, there were no RPV/TDF/FTC prescriptions for treatment-naïve patients after its approval in 2014. Eventually, RPV/TDF/FTC, which had not been claimed for reimbursements for 2 years, was deleted from the National Formulary list in 2017^24^.

Physicians favored EVG/c/TDF/FTC over RPV/TDF/FTC because of the latter’s limited and complex indications. Moreover, RPV/TDF/FTC has been associated with high virologic failure rates and drug-resistant mutations, with failures occurring among patients with baseline HIV-RNA viral loads > 100,000 copies/mL or CD4 cell counts < 200 mm^25^. On the other hand, elvitegravir (EVG) is a recently developed INSTI, and it has been recognized for its excellent efficacy and tolerability. Additionally, it is rarely associated with treatment resistance. EVG/c/TDF/FTC can be prescribed to all patients regardless of HIV-RNA levels, and thus, clinicians have selected EVG/c/TDF/FTC for various patient groups. Furthermore, RPV/TDF/FTC must be taken with at least 400 cal of food, whereas EVG/c/TDF/FTC prescriptions have no restrictions on concomitant food intake^26^. These disadvantages associated with specific ART regimens can affect the choices of clinicians who consider medication adherence and convenience.

We observed a remarkable change in ART prescription patterns after the launch of DTG/ABC/3TC and EVG/c/TAF/FTC. Dolutegravir (DTG) is a second-generation INSTI and is highly active against drug-resistant HIV strains, including strains resistant to first-generation INSTIs. DTG/ABC/3TC is recommended for once-daily treatment and can be taken more easily, with or without food^25^. EVG/c/TAF/FTC, which was approved in 2017 in Korea, reduced the adverse effects of the previously approved EVG/c/TDF/FTC. TAF is a tenofovir prodrug that results in a 90% reduction in plasma tenofovir concentrations compared with TDF, and it is, therefore, associated with fewer tenofovir-related adverse effects in the kidneys and bone^19^. As soon as DTG/3TC/ABC was approved, its prescription rate increased rapidly because of its better therapeutic effects; however, no such increase was observed for EVG/c/TAF/FTC. These findings imply that both convenience and improvements in therapeutic scope are important considerations for physicians and patients deciding on ART regimens.

STR prescription rates were higher in metropolitan areas than elsewhere between 2014 to 2016. This pattern may be due to demographic factors^27^. There is a higher proportion of young people in metropolitan areas, so new STRs, which can be taken more easily and have fewer adverse effects, may be preferred by younger patients in these areas. The general pattern of ART prescription trend changes was similar in non-metropolitan areas relative to that observed in metropolitan areas—declining MTR prescriptions and increased uptake of STR—albeit with a time lag in non-metropolitan areas.

This study had some limitations. First, we did not include clinical outcomes showing how the STRs increased patient compliance, influenced virologic suppression, or reduced tolerance. However, previous studies have demonstrated STRs are important determinants of medication adherence^28,29^. Second, we were unable to evaluate whether the ART choices were the clinically optimal regimen (i.e., in terms of opportunistic infections or renal insufficiency), as we only relied on insurance claims data for reimbursement purposes.

Despite these caveats, our data provide a valuable perspective for evaluating ART regimen prescription patterns on a national scale, compared with studies using data on ART regimens from medical records in selected clinics. Our results imply that the preferences among the single fixed-dose combination options are determined not only based on administration convenience (i.e., no requirement for concomitant food intake) but also fewer adverse effects. Although newer regimens are preferred, a new effective ingredient (DTG) was selected more frequently than an alternative component (TAF), despite the latter’s smaller adverse effect profile.

As HIV/AIDS patients get older over time, they are likely to develop comorbidities. Therefore, additional studies are warranted to clarify the relationship between medication adherence and clinical outcomes, including the adverse effects of ART medication and comorbidities, such as diabetes and dyslipidemia. We need to continue efforts to optimize ART adherence to manage HIV/AIDS as a chronic disease, particularly for patients with multiple comorbidities and psychosocial difficulties.

## Methods

### Data sources

The medical and pharmacy claims database was retrospectively obtained from the Health Insurance Review and Assessment (HIRA) service. In Korea, 97% of the total population are the mandatory beneficiaries of National Health Insurance, and the remaining 3% are recipients of the National Medical Aid program, the public assistance system for health services for people in the lowest economic bracket^30,31^. Furthermore, all medical institutions and local pharmacies are compulsory participants of the National Health Insurance and National Medical Aid programs. All medical expenditures within the National Health Insurance and National Medical Aid programs are monitored for reimbursement by the HIRA. The HIRA claims database contains basic information on patients’ sociodemographic characteristics and information on diagnoses, prescriptions, or diagnostic procedures, and it covers the entire Korean population of approximately 50 million individuals^32^.

### Study sample

**Figure 4:**
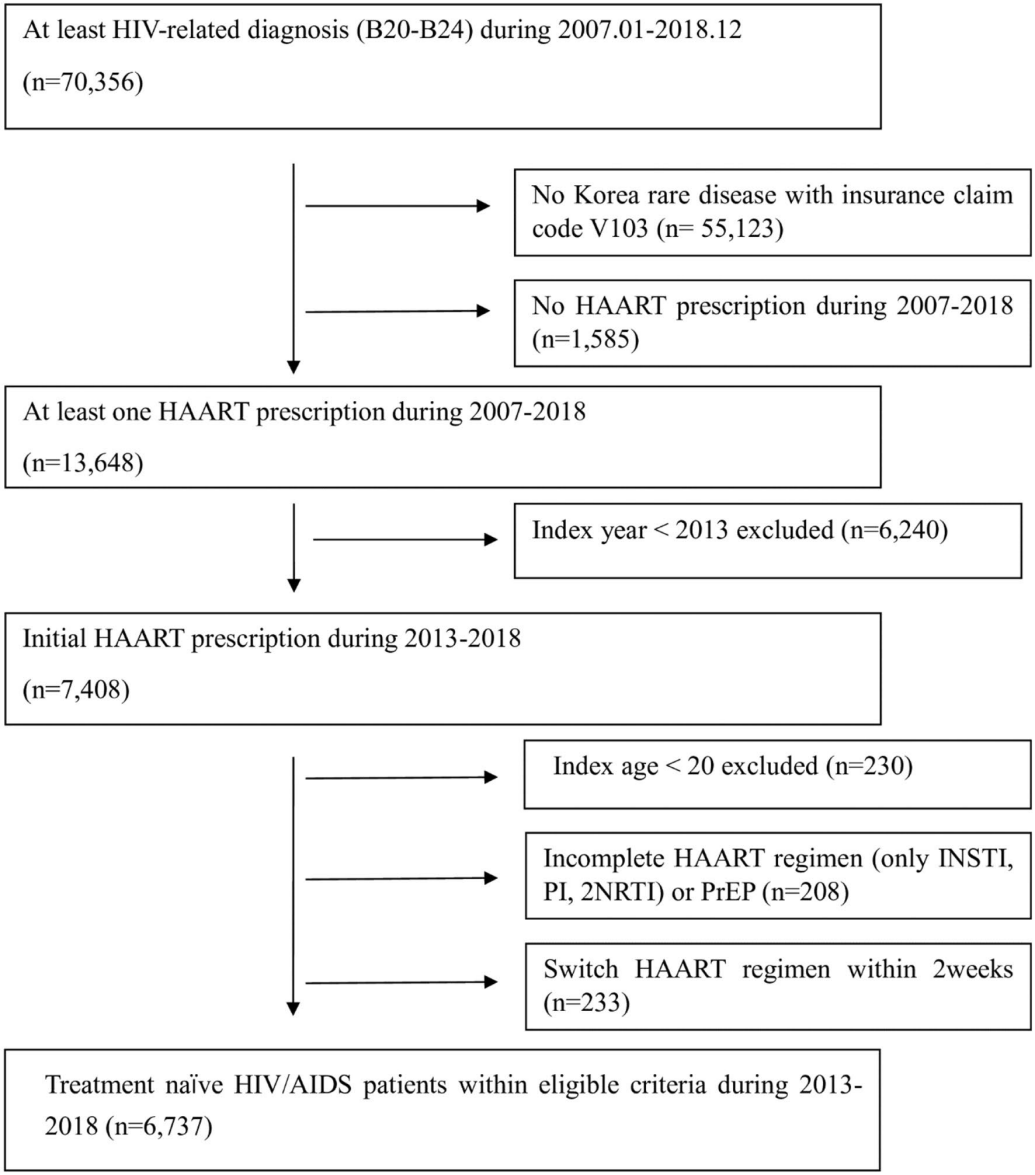
Steps for identifying the study population of treatment naïve HIV-infected patients.

Inclusion criteria were defined by HIV infection and ART prescription. First, we identified patients with HIV-related diagnostic codes B20–B24, according to the International Classification of Diseases, 10th edition (ICD-10), over 10 years, between 2007 and 2018 (n = 70,356). Second, among these, we excluded individuals who did not have a specified internal code for HIV/AIDS, indicating rare diseases, i.e., V103, in the first medical claim with an HIV/AIDS diagnosis (n = 55,123) or ART prescription (n = 1585)^33^. With the internal code in the first visit, out-of-pocket payments are waived for patients with HIV. Those who were prescribed at least 1 ART prescription during 2007–2018 (n = 13,648) remained. Third, we excluded individuals who started ART before 2013 (n = 6240), given that the first STR, EVG/c/TDF/FTC, was approved in March 2014 in Korea. Then, we excluded those under 20 years of age (n = 230), those who received ART prescriptions for pre-exposure prophylaxis (PrEP) or incomplete prescriptions (i.e., only 1 ART prescription or 2 NRTIs) (n = 208), and those who switched regimens within 2 weeks from the ART initiation (n = 233). Among the remaining 13,648 patients, 6737 patients were considered as HIV/AIDS treatment-naïve and included in the final sample for the present study (Fig. 4).

### Covariates

Sociodemographic characteristics were accessed at the initial ART prescription, which was used as the index date for each subject. The analyzed sociodemographic characteristics included sex, age, and national health insurance premium level as a proxy for income. Age at the index date was categorized by 10-year intervals starting at 20 years of age^34^. We separately identified ART initiation dates at outpatient visits and during hospital admissions. Index regimens were grouped as an STR versus MTR based on the number of pills in the regimen. Based on the treatment guidelines for initial ART administration to consist of 2 NRTIs combined with a third agent, we categorized MTR types, such as NNRTI, boosted PI or PI-based, INSTI, and NRTI-sparing (boosted protease inhibitor plus INSTI)^4,6,28^. The medical institutions were categorized as tertiary general hospitals, which are designated by government accreditation every three years based on the proportion of severe diseases versus others. Hospital locations were grouped as metropolitan areas, including the capital city, versus other regions^35^.

### Statistical analysis

All descriptive results are given as total numbers and percentages. Comparisons of percentages were performed using the χ^2^ test. All analyses were performed using Stata 15 (StataCorp, College Station, TX, USA).

### Ethics statement

This study was approved by the Institutional Review Board of Yonsei University, which waived the requirement for informed consent due to the study’s use of existing secondary claims data obtained from HIRA (approval number: 7001988-202003-HR-829-01E). The study was performed in accordance with relevant guidelines and regulations.

## Supplementary Information

Supplementary Figure 1.

Supplementary Figure 2.

Supplementary Table 1.

Supplementary Legends.

## References

[1] Althoff KN (2012). U.S. trends in antiretroviral therapy use, HIV RNA plasma viral loads, and CD4 T-lymphocyte cell counts among HIV-infected persons, 2000 to 2008. Ann. Intern. Med..

[2] HIV/AIDS, J. U. N. P. O. 90–90–90: An ambitious treatment target to help end the AIDS epidemic (UNAIDS, 2014).

[3] Ryom L (2018). Highlights of the 2017 European AIDS Clinical Society (EACS) Guidelines for the treatment of adult HIV-positive persons version 9.0. HIV Med..

[4] Günthard HF, Saag MS, Benson CA (2016). Antiretroviral drugs for treatment and prevention of HIV infection in adults: 2016 recommendations of the International Antiviral Society–USA panel. JAMA.

[5] Thompson MA (2010). Antiretroviral treatment of adult HIV infection: 2010 recommendations of the International AIDS Society–USA panel. JAMA.

[6] Thompson MA (2012). Antiretroviral treatment of adult HIV infection: 2012 recommendations of the International Antiviral Society–USA panel. JAMA.

[7] World Health Organization (2003). Adherence to Long-Term Therapies: Evidence for Action.

[8] Saag MS (2018). Antiretroviral drugs for treatment and prevention of HIV infection in adults: 2018 recommendations of the International Antiviral Society-USA panel. JAMA.

[9] Abara WE, Adekeye OA, Xu J, Rust G (2017). Adherence to combination antiretroviral treatment and clinical outcomes in a Medicaid sample of older HIV-infected adults. AIDS Care.

[10] Cotte L (2017). Effectiveness and tolerance of single tablet versus once daily multiple tablet regimens as first-line antiretroviral therapy—Results from a large French multicenter cohort study. PLoS One.

[11] Hemmige V, Flash CA, Carter J, Giordano TP, Zerai T (2018). Single tablet HIV regimens facilitate virologic suppression and retention in care among treatment naive patients. AIDS Care.

[12] Hanna DB (2014). Increase in single-tablet regimen use and associated improvements in adherence-related outcomes in HIV-infected women. J. Acquir. Immune Defic. Syndr..

[13] Cohen CJ, Meyers JL, Davis KL (2013). Association between daily antiretroviral pill burden and treatment adherence, hospitalisation risk, and other healthcare utilisation and costs in a US medicaid population with HIV. BMJ Open.

[14] Scott Sutton S, Magagnoli J, Hardin JW (2016). Impact of pill burden on adherence, risk of hospitalization, and viral suppression in patients with HIV infection and AIDS receiving antiretroviral therapy. Pharmacotherapy.

[15] Armstrong B, Chan DJ, Stewart MJ, Fagan D, Smith D (2015). Single tablet regimen usage and efficacy in the treatment of HIV infection in Australia. AIDS Res Treat.

[16] Gallant JE (2006). Tenofovir DF, emtricitabine, and efavirenz vs. zidovudine, lamivudine, and efavirenz for HIV. N. Engl. J. Med..

[17] Walmsley SL (2013). Dolutegravir plus abacavir-lamivudine for the treatment of HIV-1 infection. N. Engl. J. Med..

[18] Sax PE (2012). Co-formulated elvitegravir, cobicistat, emtricitabine, and tenofovir versus co-formulated efavirenz, emtricitabine, and tenofovir for initial treatment of HIV-1 infection: A randomised, double-blind, phase 3 trial, analysis of results after 48 weeks. Lancet.

[19] Sax PE (2015). Tenofovir alafenamide versus tenofovir disoproxil fumarate, coformulated with elvitegravir, cobicistat, and emtricitabine, for initial treatment of HIV-1 infection: Two randomised, double-blind, phase 3, non-inferiority trials. Lancet.

[20] Choi BY (2018). Korea HIV/AIDS Cohort Study: Study design and baseline characteristics. Epidemiol. Health.

[21] Korean Society for AIDS (2013). The 2013 clinical guidelines for the diagnosis and treatment of HIV/AIDS in HIV-infected Koreans. Infect. Chemother..

[22] Dharan NJ (2019). HIV treatment regimens and adherence to national guidelines in Australia: An analysis of dispensing data from the Australian pharmaceutical benefits scheme. BMC Public Health.

[23] Magagnoli J, Sutton SS, Hardin JW, Edun B (2019). Longitudinal trends in base antiretroviral therapy utilization for human immunodeficiency virus from 2000 to 2016. JACCP J. Am. Coll. Clin. Pharm..

[24] Health insurance review and assessment service (HIRA). https://biz.hira.or.kr/index.do?sso=ok (Accessed 11th Nov 2020).

[25] Sebaaly JC, Kelley D (2017). Single-tablet regimens for the treatment of HIV-1 infection. Ann. Pharmacother..

[26] Complera (rilpivirine/emtricitabine/tenofovir disoproxil fumarate) [package insert] (Gilead Sciences, 2011)

[27] Statistics Korea. http://www.index.go.kr/potal/main/EachDtlPageDetail.do?idx_cd=1007 (Accessed 11th Nov 2020).

[28] Chakraborty A, Qato DM, Awadalla SS, Hershow RC, Dworkin MS (2020). Antiretroviral therapy adherence among treatment-naive HIV-infected patients. AIDS.

[29] Sweet D, Song J, Zhong Y, Signorovitch J (2014). Real-world medication persistence with single versus multiple tablet regimens for HIV-1 treatment. J. Int. AIDS Soc..

[30] Kim MJ (2017). Causes of HIV drug non-adherence in Korea: Korea HIV/AIDS cohort study, 2006–2015. Infect. Chemother..

[31] Young J (2018). Antiretroviral pill count and clinical outcomes in treatment-naive patients with HIV infection. HIV Med..

[32] Kim J-A, Yoon S, Kim L-Y, Kim D-S (2017). Towards actualizing the value potential of Korea Health Insurance Review and Assessment (HIRA) data as a resource for health research: Strengths, limitations, applications, and strategies for optimal use of HIRA data. J. Korean Med. Sci..

[33] Health Insurance Review and Assessment Service (HIRA). https://www.hira.or.kr/dummy.do?pgmid=HIRAA030057020100 (Accessed 1st Nov 2020).

[34] Yang HJ, Bang JH (2017). Factors associated with medication adherence in patients with human immunodeficiency virus in South Korea. AIDS Care.

[35] Kim J, Lee E, Park BJ, Bang JH, Lee JY (2018). Adherence to antiretroviral therapy and factors affecting low medication adherence among incident HIV-infected individuals during 2009–2016: A nationwide study. Sci. Rep..

