# Supplementary figures and images for: Effect of single tablet regimen on prescription trends for treatment-naïve patients with HIV/AIDS in Korea
Source: Sci Rep. 2022 Feb 7;12:2031. doi: 10.1038/s41598-022-06005-0 (PMC8821544; doi:10.1038/s41598-022-06005-0)

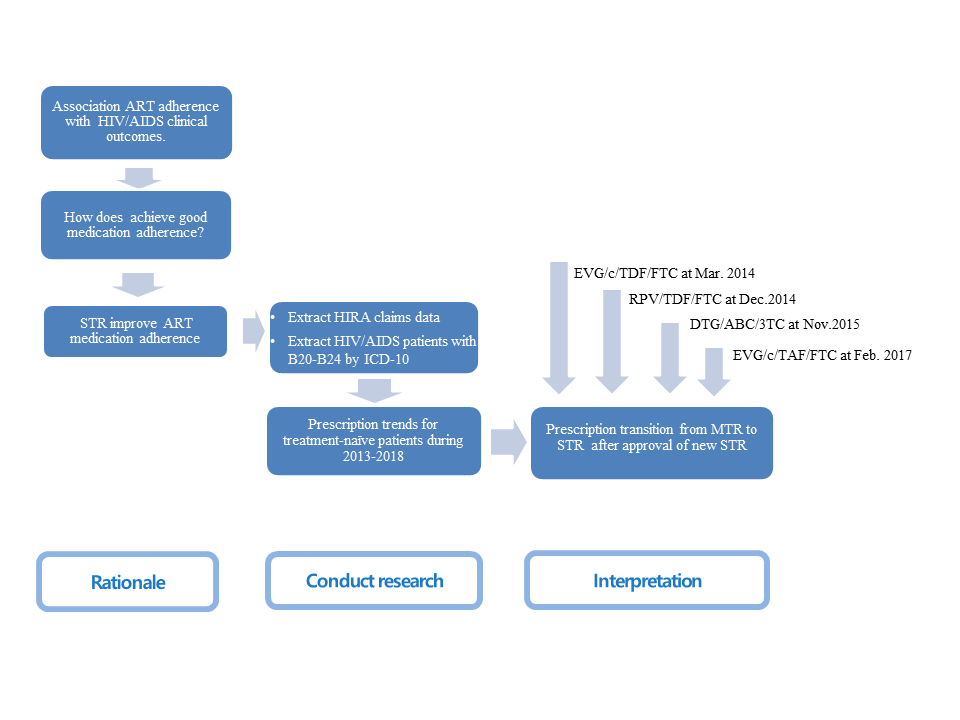

Supplement: Supplementary file 1 — Supplementary Figure 1. [file 41598_2022_6005_MOESM1_ESM.tif]

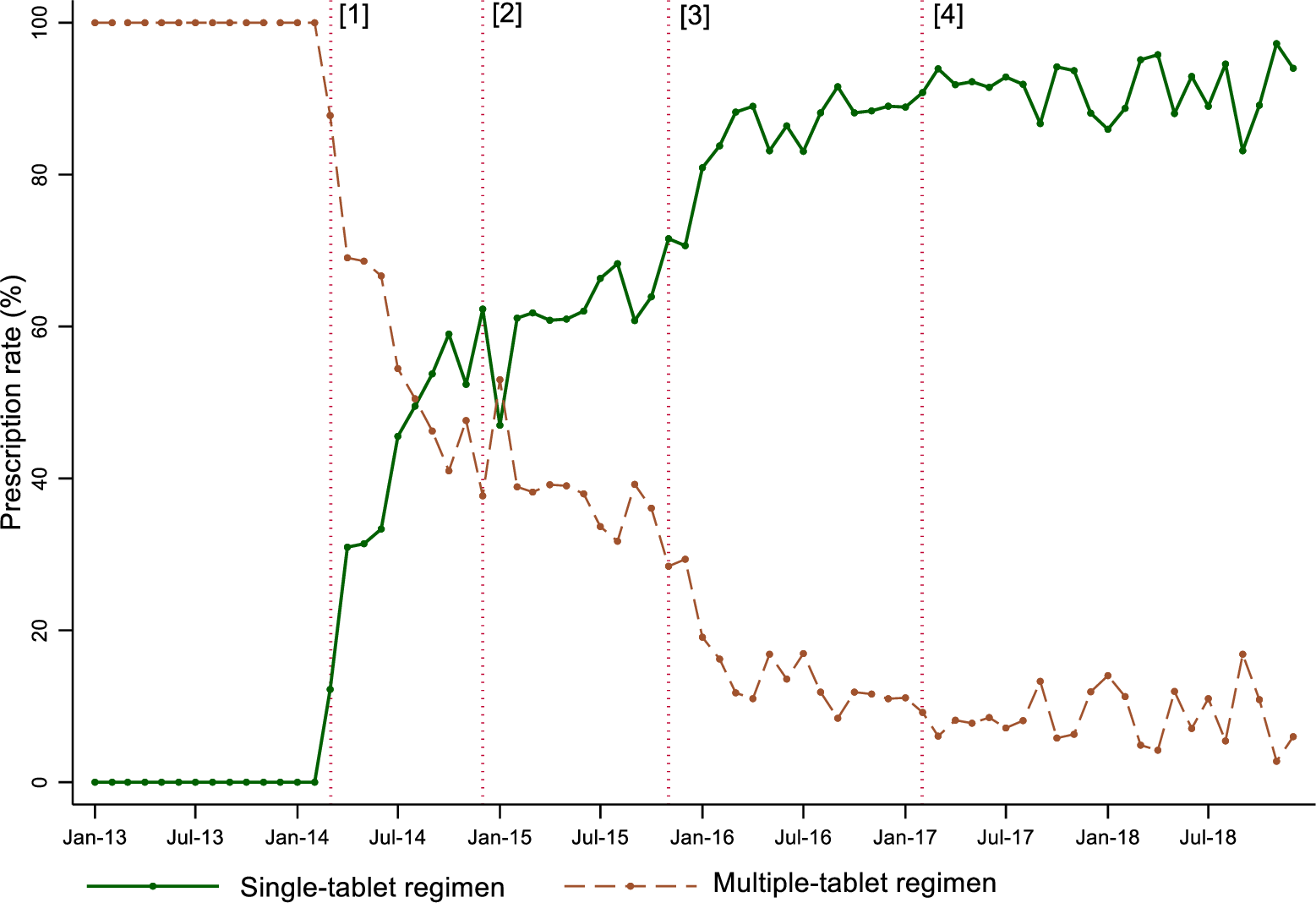

Supplement: Supplementary file 2 — Supplementary Figure 2. [file 41598_2022_6005_MOESM2_ESM.tif]
