# Supplementary material for: Effect of single tablet regimen on prescription trends for treatment-naïve patients with HIV/AIDS in Korea
Source: Sci Rep. 2022 Feb 7;12:2031. doi: 10.1038/s41598-022-06005-0 (PMC8821544; doi:10.1038/s41598-022-06005-0)
Supplement: Supplementary file 3 — Supplementary Table 1. [file 41598_2022_6005_MOESM3_ESM.pdf]

Supplementary Table 1. Newly Approved Single-Tablet Regimens for HIV/AIDS Patients in Korea

Components	ATC code	Approval date
Elvitegravir/cobicistat/Tenofovir disoproxil fumarate/emtricitabine (EVG/c/TDF/FTC)	J05AR09	Mar. 2014
Rilpivirine/Tenofovir disoproxil fumarate/emtricitabine (RPV/TDF/FTC)	J05AR08	Dec. 2014
Dolutegravir/Abacavir/Lamivudine (DTG/ABC/3TC)	J05AR13	Nov. 2015
Elvitegravir/cobicistat/Tenofovir alafenamide fumarate/emtricitabine (EVG/c/TAF/FTC)	J05AR18	Feb. 2017
