# Supplementary material for: Effect of single tablet regimen on prescription trends for treatment-naïve patients with HIV/AIDS in Korea
Source: Sci Rep. 2022 Feb 7;12:2031. doi: 10.1038/s41598-022-06005-0 (PMC8821544; doi:10.1038/s41598-022-06005-0)
Supplement: Supplementary file 4 — Supplementary Legends. [file 41598_2022_6005_MOESM4_ESM.docx]

**SUPPLEMENTARY MATERIAL**

Supplementary Figure 1. Study overview

Supplementary Figure 2. Prescription rates for single-tablet regimens and multiple-tablet regimens after single-tablet regimen approval
